# Cultural Differences in How People Deal with Ridicule and Laughter: Differential Item Functioning between the Taiwanese Chinese and Canadian English Versions of the PhoPhiKat-45

**DOI:** 10.3390/ejihpe13020019

**Published:** 2023-01-20

**Authors:** Chloe Lau, Taylor Swindall, Francesca Chiesi, Lena C. Quilty, Hsueh-Chih Chen, Yu-Chen Chan, Willibald Ruch, René Proyer, Francesco Bruno, Donald H. Saklofske, Jorge Torres-Marín

**Affiliations:** 1 Centre for Addiction and Mental Health, Toronto, ON N6B 1Y6, Canada; 2 Department of Psychology, University of Western Ontario, London, ON N6A 3K7, Canada; 3 Department of Neuroscience, Psychology, Drug, and Child’s Health (NEUROFARBA), Section of Psychology, University of Florence, 50135 Florence, Italy; 4 Department of Educational Psychology and Counseling, National Taiwan Normal University, Taipei 106308, Taiwan; 5 Department of Educational Psychology and Counseling, National Tsing Hua University, Hsinchu 300044, Taiwan; 6 Department of Psychology, University of Zurich, 8006 Zurich, Switzerland; 7 Institut für Psychologie, Martin-Luther-Universität Halle-Wittenberg, 06108 Halle, Germany; 8 Regional Neurogenetic Centre (CRN), Department of Primary Care, ASP Catanzaro, Viale A. Perugini, 88046 Lamezia Terme, Italy; 9 Association for Neurogenetic Research (ARN), 88046 Lamezia Terme, Italy; 10 Academy of Cognitive Behavioral Sciences of Calabria (ASCoC), 88046 Lamezia Terme, Italy; 11 Department of Social Psychology and Quantitative Psychology, University of Barcelona, 08035 Barcelona, Spain; 12 Department of Research Methods in Behavioral Sciences, University of Granada, 18071 Granada, Spain

**Keywords:** laughter, ridicule, humour, cross-cultural differences, differential item functioning, gelotophobia, gelotophilia, katagelasticism

## Abstract

The PhoPhiKat-45 measures three dispositions toward ridicule and laughter, including gelotophobia (i.e., the fear of being laughed at), gelotophilia (i.e., the joy of being laughed at), and katagelasticism (i.e., the joy of laughing at others). Despite numerous cultural adaptations, there is a paucity of cross-cultural studies investigating measurement invariance of this measure. Undergraduate students from a Canadian university (*N* = 1467; 71.4% females) and 14 universities in Taiwan (*N* = 1274; 64.6% females) completed the English and Chinese PhoPhiKat-45 measures, respectively. Item response theory and differential item functioning analyses demonstrated that most items were well-distributed across the latent continuum. Five of 45 items were flagged for DIF, but all values had negligible effect sizes (McFadden’s pseudo *R*^2^ < 0.13). The Canadian sample was further subdivided into subsamples who identified as European White born in Canada (*n* = 567) and Chinese born in China, Hong Kong, or Taiwan (*n* = 180). In the subgroup analyses, no evidence of DIF was found. Findings support the utility of this measure across these languages and samples.

## 1. Introduction

Humour is a multidimensional phenomenon that encompasses a function (e.g., pro-social or mean-spirited) and fulfills complex needs for the individual (e.g., engage with others, mock others) [1]. Ruch [1] proposed that humour measures should have both humour and humourlessness represented, such that some individuals enjoy humorous interactions while others do not and thus avoid it. In response, Ruch and Proyer [2] proposed the measurement of three dispositions toward ridicule and laughter, including gelotophobia, gelotophilia, and katagelasticism. Gelotophobia is defined as the fear of being laughed at, a construct theoretically distinct from social anxiety, in which good-natured laughter may be perceived as a threatening form of ridicule directed towards the target [3,4]. Gelotophobia is highly correlated with fear of social situations, feeling perturbed, and greater stress in the workplace [5]. In contrast to the clinical categorization, a gelotophobic response is distinguished by an increased sensitivity to laughter [5]. Gelotophilia is the joy of being laughed at, in which joint laughter is perceived as a sign of appreciation [2]. Individuals who are high in gelotophilia do not mind being laughed at, as they see the role such as “class clown” desirable. Katagelasticism is the joy of laughing at others, in which the individual high in this trait may feel entitled to laugh at others at their expense if the opportunity presents itself [2]. 

In terms of measurement, these dispositions are commonly measured using the self-report instrument named PhoPhiKat (for long 45/30 item-version see Ruch and Proyer [2]; for ultrashort 9 item-version see Hofmann et al. [5]). The PhoPhiKat-45 is a self-report measure with supported structural, convergent, and discriminant validity [6,7]. Since its initial empirical validation in German-speaking samples [2], this measure has been translated and adapted in various languages, including Chinese, English, Turkish, Russian, and Spanish [7,8,9,10,11,12]. 

*The PhoPhiKat across cultures*. Though prior adaptations of the PhoPhiKat questionnaire are solid contributions to the scientific literature, none of these studies have investigated the cross-cultural validity of this instrument. One might wonder, for instance, whether the information derived from the PhoPhiKat scores might be equally generalized to Eastern and Western countries. The psychometric properties of a revised PhoPhiKat questionnaire for Chinese individuals have been supported across several studies [8,13], where it demonstrated strong construct and structural validity, internal consistency, and test-retest reliability. Similarly, Chen et al. [8] showed promising results for the reliability and validity of the traditional Chinese version, titled PhoPhiKat-TC. Translated versions of the PhoPhiKat were translated from English, to a different language, and then back to English for further analysis [8]. These measures were developed to enable: (1) global psychological investigation and experimental design with psychometrically sound tools; and (2) cross-cultural comparisons between individualistic and collectivistic cultures in order to further study cultural differences in humour and humour-related traits [8].

In the United States, the English version has been utilized in the cross-cultural study of gelotophobia in particular [14,15,16]. With focus on European and Asian Americans, Asian Americans scored higher compared to other cultures on the gelotophobia factor, which was interpreted as a result of the societal norm of collectivism among Asian cultures [8,15,17]. It is critical that measurement equivalence is established across cultures prior to concluding that the overall scale and its individual items have the same meaning across cross-cultural samples, however [18,19,20]. Measurement invariance analyses provide evidence that differences in test scores reflect true latent variable differences than group differences based on measurement bias [21,22]. The difference among cultural norms within a similar geographical location provide rationale for further cross-cultural research on the potential psychometric and observed means-differences in the PhoPhiKat-45.

*How culture shapes humour and laughter*. The presence of humour is commonplace, however, it may be perceived in different ways depending on one’s cultural norms [23]. As outlined in Jiang, Li, and Hou [23], being humorous is viewed as a positive character trait and often used in everyday life in Western cultures. In Eastern cultures, it is largely not viewed as positively, despite occasional utilization to promote belongingness in attempt to raise social status [23,24]. Numerous studies have demonstrated how humour characteristics (i.e., appreciation, production, correlates) can vary among cultures such as Hong Kong and Taiwan [25,26], pushing forward its importance for further study. 

Established in Taiwanese and Chinese samples, previous studies found that those among a collectivistic culture experience humour differently, with Taiwanese and Chinese individuals reporting to be more afraid of being laughed at due to the threat of group harmony [27,28]. Comparatively, as demonstrated in European and North American samples, those in individualistic societies may express less fear of being laughed at as it would not pose a threat to a group environment [28]. As Chinese culture stresses conformity and withholding of particular emotional reactions, aligned with the collectivist view, this information is not unprecedented, similarly to the results for the individualistic [28,29].

Amidst individuals with gelotophobia, there is greater avoidance of social situations with laughter-related emotions, comparable to previous findings revealing evidence associating gelotophobia with low extraversion [28,30,31]. Gelotophobia was linked to a lower life of engagement in a cross-cultural comparison, in addition to a lower life of meaning and pleasure among those in China, which lastly aids the explanation of why some aspects of humour-related emotions and traits are often avoided [11]. Concentrating on gelotophilia, those who are high in this dimension may ‘expose’ themselves to get laughed at, as demonstrated by English North Americans within the same study [28,31]. The comparison between Chinese individuals and English North Americans is important to understand how cultures can vary within as well as across geographical regions [32].

In summary, there are differences within and across cultures in how individuals perceive aspects of humour and humour-related traits. This distinction appears greatest between individualistic and collectivistic cultures, and may determine how a particular cultural group may define humour and its associations with key outcomes [32]. Further, there is stronger background on cross-cultural research relating to gelotophobia, with less information on gelotophilia and katagelasticism. The current investigation undertakes to further extend this literature, and to respond to this important evidence gap, in an evaluation of differences among these factors and their associations with perceptions and cultural beliefs across cultural groups.

*The present research*. Presently, there are a lack of cross-cultural studies on the PhoPhiKat-45 measure. The current study will respond by using item response theory (IRT) and differential item functioning (DIF) to be the first to investigate the psychometric properties of the measure and its relation to different samples. The present study aims to investigate: (1) the item response parameterization of the English and Chinese version of the PhoPhiKat-45; (2) evaluate uniform and non-uniform DIF for individual items with these two groups; and (3) further subdivide Canadian individuals to those who identified as European White and Chinese to evaluate whether DIF represents differing cultural perceptions within the same geographical region. 

The first objective employs IRT, a technique to determine how much information a particular item on a measure or questionnaire may provide to the researcher [29,33,34,35,36,37]. In using an item characteristic curve to determine the relationship between a participant and a given item, IRT can define the precision of items in both the English and Chinese measures. DIF is often utilized to interpret differences between groups within a measure in a meaningful way, such as determining group differences [34,38,39]. When considering previous works, it is hypothesized that the Chinese sample may have a higher likelihood of selecting an answer high in gelotophobia in comparison to the English sample based on measurement biases rather than cultural differences [15,17]. Thus, questions can be asked about biases due to cultural differences, similar to Lampert, Isaacson, and Lyttle’s [15] study, or if another inference can be made. DIF can threaten test validity and highlight the need to thoroughly investigate each measure and account for any dangers to its strength [37,38,40,41,42,43,44]. By employing IRT and DIF in the present study, the aim is to provide evidence for the psychometric properties and cross-cultural measurement invariance of the PhoPhiKat-45 in English and Chinese.

## 2. Materials and Methods

### 2.1. Participants

In the Canadian sample, undergraduate students (*N* = 1467; 71.4% females) participated in the study online through the undergraduate participant research recruitment pool. Qualtrics, a web-based survey tool, was used for anonymized data collection. The sample identified as European White (*n* = 610; 41.6%), Asian/Pacific Islander (*n* = 592; 40.4%), Black/African American (*n* = 38; 2.6%), Hispanic/Latino (*n* = 10; 0.7%), Native American (*n* = 2, 0.1%), and other identity or prefer not to say (*n* = 215; 14.7%). Students’ ages ranged from 16 to 54 years (*M* = 18.36, *SD* = 1.68). Study participation was voluntary, and participants received a credit towards a psychology course. Participants were provided with informed consent and debriefed.

In the Taiwanese Chinese sample, data were collected using paper and pencil from undergraduate students from 14 universities located in Northern, Central, Southern, and Eastern areas in Taiwan (*N* = 1274; 64.6% females) between the ages of 18 to 29 years (*M*_age_ = 19.74; *SD* = 1.48). Students participated voluntarily and were debriefed after participation. 

### 2.2. Materials and Procedure

**Figure 1:**
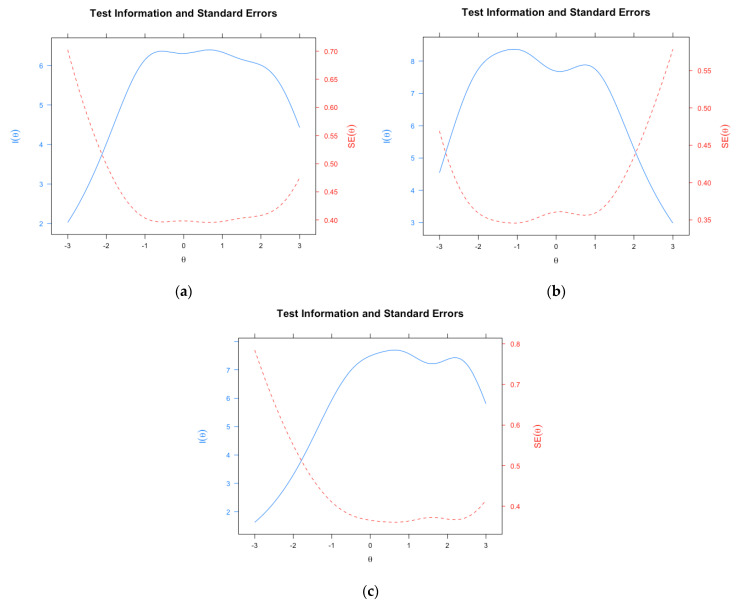
Phophikat-45 test information function for the Canadian Sample. Graphs from left to right depict (**a**) gelotophobia, (**b**) gelotophilia, and (**c**) katagelasticism, respectively, in the Canadian Sample. I represent information and θ represents latent trait. SE represents standard error.

**Figure 2:**
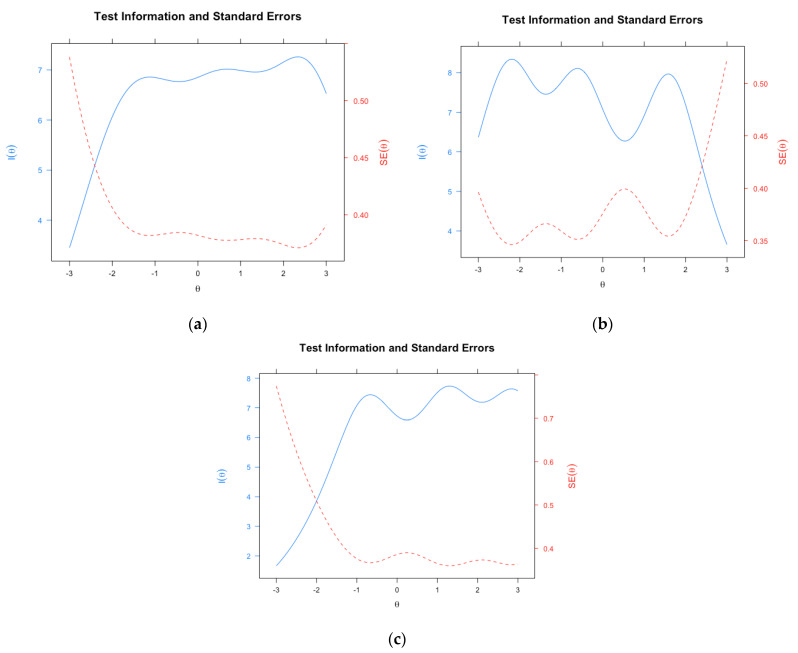
Phophikat-45 test information function for the Taiwanese Chinese Sample. Graphs from left to right depict (**a**) gelotophobia, (**b**) gelotophilia, and (**c**) katagelasticism, respectively, in the Taiwanese Sample. I represent information and θ represents latent trait. SE represents standard error.

*PhoPhiKat-45.* The PhoPhiKat-45 is a reliable and valid measure with 45 items that assesses gelotophobia (i.e., the fear of being laughed at), gelotophilia (i.e., the joy of being laughed at), and katagelasticism (i.e., the joy of laughing at others) [4,45,46]. Each respondent rates the items on a four-point Likert type scale ranging from *strongly disagree* to *strongly agree*. The measure has demonstrated evidence of strong internal consistency, test-retest reliability, and structural, convergent, and discriminant validity [4,5,8,45,46]. For the English version, Bayesian single-test reliability values with 95% credible intervals (CIs) of posterior means determined by MacDonald’s Omega values for gelotophobia, gelotophilia, and katagelasticism were 0.84 (0.82,0.85), 0.86 (0.85,0.87), and 0.84 (0.83,0.85), respectively. For the Taiwanese Chinese version, Bayesian MacDonald’s Omega values for posterior means were 0.85 (0.84,0.86), 0.84 (0.83,0.86), and 0.84 (0.83,0.85), respectively. Test information functions (TIF) are presented in Figure 1 and Figure 2 (see results) for the Canadian English and Taiwanese Chinese versions, respectively. Note that “I” represents information in the figures. 

### 2.3. Analytic Strategy

The marginal maximum likelihood estimation method was used to evaluate gelotophobia, gelotophilia, and katagelasticism through Samejima’s graded response model (GRM) [47]. Orlando and Thissen’s [48] S-*X*^2^ statistics were reported for item fit [49]. Local dependence (LD) is evaluated using Yen’s *Q*_3_ statistics for excess item covariation when the latent trait is controlled for [50]. Marais and Andrich [51] recommended critical residual correlation values between 0.10 to 0.30. For this study, a value of 0.30 was used to test the violation of the local independence assumption. Yen’s *Q*_3_ values showed no evidence for the violation of the assumption of local item independence for all items in the Canadian English Sample. In the Taiwanese Chinese sample, most items showed no evidence for the violation of the assumption of local item independence; however, items 1 and 7 showed a residual correlation of 0.46. Specifically, these two items shared overlap in content regarding being paranoid about being the person laughed at when overhearing strangers laughing. As such, method variance may lead to residual correlations that the latent trait cannot explain [52]. 

In Samejima’s graded response model, a single item discrimination value (*a*) and three category threshold (*b*_i_) function values were produced for every item. Discrimination values were categorized as follows: ≤0.24 as very low, 0.25 to 0.64 as low, 0.65 to 1.34 as moderate, 1.35 to 1.69 as high, and ≥1.7 as very high [53]. The threshold parameters (*b_i_*) were presented as *z*-scores (*M* = 0, *SD* = 1) and indicate the amount of latent trait required for a 50% probability of endorsing the following response category.

DIF was used to determine whether biases across the Canadian English and Taiwanese Chinese versions existed. Crane et al. [54] recommended an ordinal regression approach to DIF that follows Samejima’s [47] graded response model (GRM) [55,56]. Based on the latent trait, each item incorporates three separate ordinal regression models. Model 1 (scores) includes the model at baseline with response probabilities regressed on the latent trait. Model 2 (scores and group) is a uniform DIF model with the main effect specified by group. Model 3 is a non-uniform DIF model that includes an interaction term between the participants’ latent trait and group-specific covariate [57]. Uniform DIF is identified with the comparisons of models 1 and 2 and non-uniform DIF is established with comparisons between models 2 and 3 [57]. Total DIF effects are established with comparisons between models 1 and 3 [54,58,59]. 

To establish statistically significant DIF, distinctive criteria with differing sensitivity have been proposed [57]. According to Jeong and Lee [60], McFadden’s pseudo *R*^2^ statistic fits Kvalseth’s eight criteria for a reliable *R*^2^ [60,61,62]. With previous findings supporting the oversensitivity of χ^2^ approaches in DIF detection, the threshold of McFadden’s pseudo *R*^2^ > 0.035 was used to examine DIF [59]. Jeong and Lee’s [60] recommendations for DIF effect sizes for *R*^2^ are as follows: <0.13 as negligible, 0.13 to 0.26 as moderate, and >0.26 as large [59,60]. A 10% change in proportional β represents a meaningful size of uniform DIF [54,63]. Bayesian reliabilities were conducted on JASP version 0.16.3 [64,65]. Samejima’s GRM models and DIF were conducted on R version 4.2.1 packages *mirt* [66] and *lordif* [57]. 

## 3. Results

### 3.1. Item Response Theory 

Unidimensionality for each of the gelotophobia, gelotophilia, and katagelasticism dimensions was established, such that the first factor should account for >20% of the variance met by both samples [39]. However, the Canadian English sample shows a negative slope for item 13 (i.e., I believe that I make involuntarily a funny impression on others), suggesting misinterpretation for the translation of this item from the original German measure. Thus, it was removed from subsequent analyses [45]. For the Canadian sample, discrimination parameters for the three subscales are as follows: gelotophobia Mdn(a) = 1.18 (Min = 0.77, Max = 1.73), gelotophilia Mdn(a) = 1.29 (Min = 0.80, Max = 2.28), katagelasticism Mdn(a) = 1.29 (Min = 0.63, Max = 2.14). Similarly, discrimination parameters for the three subscales in the Taiwanese Chinese sample are as follows: gelotophobia Mdn(a) = 1.34 (Min = 0.81, Max = 1.91), gelotophilia Mdn(a) = 1.41 (Min = 0.62, Max = 2.55), katagelasticism Mdn(a) = 1.31 (Min = 0.59, Max = 2.12). Details regarding the discrimination and threshold parameters for each item are provided in Appendix A. 

### 3.2. Test Information Function (TIF)

The evaluation of TIFs for the Canadian English (Figure 1) and Taiwanese Chinese (Figure 2) samples showed similar measurement precision across the latent trait (i.e., θ). In the Canadian sample, the information and associated standard errors of measurement (SEs) showed precise measurement for respondents falling approximately within −1.5 to +3.0, −2.0 to +1.5, and −1.0 to +3.0 of the gelotophobia, gelotophilia, and katagelasticism dimensions, respectively, in the latent trait continuum. This is indicated by the maximum I and minimum SE (Figure 1 and Figure 2 for the graphical representation of the TIF). In the Taiwanese Chinese sample, the TIF for gelotophobia, gelotophilia, and katagelasticism showed precise measurement of θ from roughly −1.5 to +3.0, −3.0 to +1.5, and −1.5 to +3.0, respectively, as evidenced by the maximum I and minimum SE.

### 3.3. DIF Subgroup Analyses

To ascertain whether DIF biases existed when Chinese participants completed the English version of the PhoPhiKat-45, subgroup analyses were conducted within the English Canadian sample. This group was further divided into a group who identified as European White born in Canada (N = 567; 77.4% females; M_age_ = 18.40; SD = 1.98) and a group who identified as Chinese born in China, Taiwan, or Hong Kong (N = 180; 72.8% females; M_age_ = 18.73, SD = 1.17). The same criterion threshold of McFadden’s R^2^ > 0.035 and 10% change in proportional β was used to examine DIF. No significant DIF was identified across the three subscales of gelotophobia, gelotophilia, and katagelasticism. 

### 3.4. DIF and Effect Sizes

**Figure 3:**
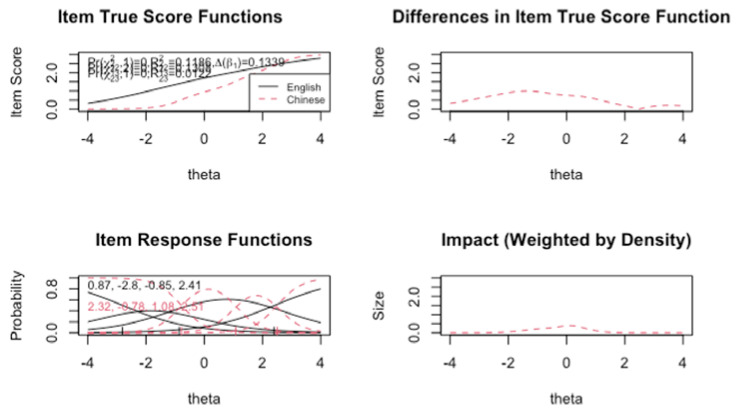
Differential item functioning in item 21 of the Katagelasticism scale. Black line represents Canadian English sample and dashed red line represents Taiwanese Chinese sample.

The results from the ordinal logistic regression for DIF detections when comparing the Canadian English and Taiwanese Chinese versions are displayed in Table 1. Among the 14 gelotophobia items, 2 (i.e., items 37 and 43) were flagged as DIF items with the McFadden’s pseudo R^2^ criterion. Among the 15 gelotophilia items, no items were flagged for DIF with the McFadden’s pseudo R^2^ criterion. Among the 15 katagelasticism items, three items were flagged for DIF with the McFadden’s pseudo R^2^ criterion. However, all values are categorized with negligible effect sizes. McFadden’s pseudo R^2^ statistics were negligible for all items (<0.13) with the largest only at 0.13 in pseudo R^2^ between Models 1 and 3 for item 21. For a proportional 10% β change, item 21 was the only item with a change greater than 10%. In the item response function of item 21, for every response category, the solid line (Canadian English) was located to the left of the corresponding dashed line (Taiwanese Chinese), suggesting that over θ, the Canadian English sample have a greater probability of selecting a higher response option than the Taiwanese Chinese sample for this particular item (Figure 3).

## 4. Discussion

The purpose of this study was to provide evidence for the psychometric properties of the PhoPhiKat-45 in English and Chinese in two independent samples collected from Canada and Taiwan. Using IRT parameters for the Canadian English and Taiwanese Chinese versions, items were well-distributed across the latent continuum and showed high discrimination parameters allowing differentiation across different levels of gelotophobia, gelotophilia, and katagelasticism. Item characteristic curves for each individual item demonstrated high discrimination parameters that were well spread across the latent continuum for gelotophobia, gelotophilia, and katagelasticism, respectively measured. Notably, one item (i.e., item 13 “*I believe that I make involuntarily a funny impression on others*”) was excluded from the gelotophobia latent trait analysis due to a negative slope identified that precluded further analyses. The English PhoPhiKat-45 measure was translated from the German version and this item may be read by English speakers as making a funny impression or appearing hilarious or amusing in front of others naturally. Although the Taiwanese Chinese version was translated from the English version, the item performed well in the Taiwanese sample. Future studies should investigate whether this item may be removed or modified for the English measure of the PhoPhiKat-45. 

The results showed that single test reliability values were acceptable for all three dimensions of gelotophobia, gelotophilia, and katagelasticism. The measurement precision of the test was evaluated using the TIF [67,68,69]. Interestingly, both the English and Chinese versions of the measure show that the scale does not capture extremely low ends of gelotophobia and katagelasticism and the extremely high end of gelotophilia. It is worth noting that both gelotophobia and katagelasticism are quasi-traits, such that both traits are unipolar constructs intended to measure only the presence or absence of the trait [2,70]. As such, the low end of the gelotophobia spectrum suggests the absence of fear of being laughed at rather than the presence of gelotophilia. These findings corroborate Ruch and Proyer’s [2] initial conceptualization of gelotophobia, that it is not a bipolar trait which would entail both extreme ends of the spectrum as separate entities (i.e., gelotophobia and gelotophilia). These results suggest that the absence of gelotophobia (i.e., no fear of being laughed at) does not indicate presence of gelotophilia (i.e., the joy of being laughed at). As such, both measures are needed for accurate measurement of these separate constructs.

Measurement invariance using DIF was also conducted to investigate whether meaningful interpretation of mean group comparisons may be made without significant biases. At the single-item level, five items were flagged for DIF in the Taiwanese Chinese version against the original English version. Only one item (i.e., item 21 *“some people set themselves up for one to make fun at them”*) analyzed has 10% change in β and over 0.13 in McFadden’s pseudo *R*^2^. Following an approach by Lau, Chiesi, and Saklofske [32] and Li et al. [71], the Canadian sample who completed the measure was subdivided into those who identified as European White born in Canada and those who identified as Chinese born in China, Hong Kong, or Taiwan. In the subgroup analyses, no evidence for DIF was found, suggesting that the DIF identified in the Taiwanese Chinese and English versions are more likely linked to subtle shifts in meaning with the translation process rather than the cultural concepts covered by the item content. Cultural expressions of affect, emotions, and behaviours vary cross cultural groups, which may lead to cross-cultural comparison biases [72]. Overall, there is support for using this scale to assess gelotophobia, gelotophilia, and katagelasticism with Chinese university students in either Chinese or English languages. DIF findings were mostly negligible and do not appear to reflect attainable differences in research or clinical practice. 

Several limitations from the present investigation should be addressed in future studies. First, both the Canadian and Taiwanese samples of undergraduate university students represent young and well-educated individuals. Future research should replicate these findings to investigate whether findings are generalizable across age groups (e.g., youth, older adults), clinical status (e.g., those diagnosed with physical illnesses or mental health disorders), and socioeconomic background. Second, although the present study included an extensive assessment of individual item performance of the PhoPhiKat-45, future studies will benefit from investigating whether these variables have the same predictive validity across cultures. Specifically, Chinese individuals conceptualize the value of humour in everyday life differently than those from Western cultures given the early historical and cultural influences of Confucianism, Taoism, and Buddhism (for a review see Yue [17]). With Chinese individuals respecting authority and seniority more than Canadians, Chinese individuals tend to be more careful and conservative when using humour [17]. Whereas a serious disposition may be associated with lower well-being in Western cultures, seriousness is positively associated with well-being in China [73,74,75,76]. For example, Singaporean students in Nevo, Nevo, and Yin’s [77] study represented more conventional traits in relation to jokes and humour, such as aggressiveness, contrast to students in the United States, who joked more about sexual items. In addition, they did not use humour as a way to cope unlike other countries studied, such as Israel, demonstrating that a culture’s values can impact an individual’s humour [77]. With this in mind, future studies should examine whether some levels of gelotophobia may be protective for the individual in a culture where humour may be avoided or seen as distasteful [17,78]. Lastly, the present study reported the proportion of DIF items that may affect group means, but there are other methods such as the reporting of linking errors (i.e., robust linking methods [79]) and DIF variance. Future studies should explore these avenues to enable the assessment of systematic biases between countries. 

## 5. Conclusions

Overall, IRT and DIF analyses conducted on the English and Taiwanese Chinese versions of the PhoPhiKat-45 demonstrated strong psychometric properties for assessing gelotophobia, gelotophilia, and katagelasticism. With the exception of one item, cross-cultural comparisons of the measure did not present biases with significant effect sizes for the individual item characteristics. Through a subgroup analysis, it is suggested that translations may alter item meanings rather than cultural concepts. However, variance across cross cultural groups may result in comparison biases, such as differences in the conceptualization of humour, including cultural influences, trait representation, and personal coping methods. Findings from this investigation support the utility of the PhoPhiKat-45 English and Chinese versions, and support the generalizability of gelotophobia, gelotophilia, and katagelasticism measures across different languages.

## Figures and Tables

**Table 1:** Likelihood ratio χ^2^ statistics, McFadden’s pseudo *R*^2^, and change in β between Canadian and Taiwanese samples.

Item	Content	χ^2^ Statistic			Δ β_1_ (%)	McFadden’s Pseudo *R*^2^			DIF Identified?
Gelotophobia Scale		χ _12_	χ _13_	χ _23_	Beta_12_	McFadden_12_	McFadden_13_	McFadden_23_	
1	When they laugh in my presence I get suspicious.	<0.001	<0.001	0.1548	3.59	0.0154	0.0157	0.0003	No
4	I avoid displaying myself in public because I fear that people could become aware of my insecurity and could make fun of me.	<0.001	<0.001	0.0662	4.30	0.0165	0.0170	0.0005	No
7	When strangers laugh in my presence I often relate it to me personally.	<0.001	0.0030	0.5758	0.27	0.0018	0.0018	<0.0001	No
10	When others make joking remarks about me I feel being paralyzed.	<0.001	<0.001	<0.001	4.63	0.0187	0.0221	0.0034	No
16	I control myself strongly in order not to attract negative attention so I do not make a ridiculous impression.	<0.001	<0.001	<0.001	0.41	0.0160	0.0214	0.0054	No
19	When I have made an embarrassing impression somewhere, I avoid the place thereafter.	<0.001	<0.001	0.3272	0.10	0.0036	0.0037	0.0001	No
22	If someone has teased me in the past I cannot deal freely with him forever.	<0.001	<0.001	0.0026	0.34	0.0020	0.0036	0.0016	No
25	It takes me very long to recover from having been laughed at.	0.1872	<0.001	<0.001	0.28	0.0003	0.0025	0.0022	No
28	Especially when I feel relatively unconcerned, the risk is high for me to attract negative attention and appear peculiar to others.	0.6403	0.7858	0.6075	0.10	<0.0001	0.0001	<0.0001	No
31	It is difficult for me to hold eye contact because I fear being assessed in a disparaging way.	<0.001	<0.001	0.5111	0.19	0.0040	0.0041	0.0001	No
34	Although I frequently feel lonely, I have the tendency not to share social activities in order to protect myself from derision.	<0.001	<0.001	0.0439	0.01	0.0086	0.0092	0.0006	No
37	When I have made a fool of myself in front of others I grow completely stiff and lose my ability to behave adequately.	<0.001	<0.001	0.0278	9.89	0.0445	0.0452	0.0008	Yes
40	While dancing I feel uneasy because I am convinced that those watching me assess me as being ridiculous.	0.1849	0.3846	0.6954	0.18	0.0002	0.0003	<0.0001	No
43	If I did not fear making a fool of myself I would speak much more in public.	<0.001	<0.001	<0.001	4.94	0.0398	0.0459	0.0062	Yes
Gelotophilia									
2	When I am with other people, I enjoy making jokes at my own expense to make the others laugh.	<0.001	<0.001	0.5386	3.10	0.0254	0.0254	0.0001	No
5	I do not hesitate telling friends or acquaintances something embarrassing or a misfortune that happened to me, even at the risk of being laughed at.	0.2941	0.3687	0.3442	0.07	0.0002	0.0003	0.0001	No
8	There is no difference for me whether people laugh at me or laugh with me.	<0.001	<0.001	0.0018	2.19	0.0117	0.0132	0.0015	No
11	I enjoy it if other people laugh at me.	<0.001	<0.001	0.0028	4.30	0.0237	0.0251	0.0014	No
14	I am the joker in my circle of friends, who entertains the others (often with jokes at my own expense).	<0.001	<0.001	0.5087	1.03	0.0044	0.0045	0.0001	No
17	I enjoy it if other people poke fun at me since this might also be a sign of recognition.	0.0037	0.0031	0.0767	0.34	0.0014	0.0019	0.0005	No
20	If someone caught me on a camera while something embarrassing or a misfortune happen to me, I would not mind, if s/he send the tape to a television show that broadcast such videos.	0.1197	<0.001	<0.001	0.21	0.0004	0.0039	0.0035	No
23	I have talent for being a comedian, cabaret artist or clown.	<0.001	<0.001	0.0843	1.85	0.0079	0.0083	0.0004	No
26	For raising laughs I pleasurably make the most out of embarrassments or misfortunes that happen to me which other people would be ashamed of.	0.0486	<0.001	<0.001	0.16	0.0006	0.0034	0.0028	No
29	I enjoy contributing to the open laughter of others by telling them embarrassing things or misfortunes that happened to me.	<0.001	<0.001	0.5934	3.83	0.0207	0.0208	<0.0001	No
32	When I am with other people and something embarrassing happens to me (e.g., a slip of the tongue or a misfortune) I am more pleased than angry and laugh along with it.	<0.001	<0.001	0.8739	1.24	0.0065	0.0065	<0.0001	No
35	If I drop a clanger, I enjoy it a little because I can hardly wait to tell my friends about this misfortune.	<0.001	<0.001	<0.001	0.51	0.0030	0.0139	0.0109	No
38	I do not mind telling something embarrassing in a group that happened to me if I know that the others will find it funny.	<0.001	<0.001	0.4553	4.91	0.0272	0.0273	0.0001	No
41	Nothing much could happen to me that I would be so ashamed that I would not tell it others.	<0.001	<0.001	0.0007	2.67	0.0142	0.0159	0.0017	No
44	My friends know me for not being ashamed of telling them of embarrassing situations that happened to me.	<0.001	<0.001	<0.001	0.26	0.0049	0.0109	0.0059	No
Katagelasticism									
3	I enjoy exposing others and I am happy when they get laughed at.	0.0747	0.1397	0.3829	0.42	0.0005	0.0006	0.0001	No
6	Often, disputes emerged because of funny remarks or jokes that I make about other people.	<0.001	<0.001	0.0390	3.55	0.0117	0.0125	0.0007	No
9	When related to making jokes or funny remarks about other people I rather follow the motto ‘‘An eye for an eye, a tooth for a tooth’’ than ‘‘If someone strikes you on the right cheek, offer him the other also.’’	0.5718	0.0054	0.0015	0.15	<0.0001	0.0016	0.0015	No
12	It has happened that humorless persons have broken o¤ their friendship with me or at least threatened me to do so, because I overdid ridiculing them over of something embarrassing or a misfortune that happened to them.	<0.001	<0.001	<0.001	1.96	0.0034	0.0068	0.0034	No
15	If other people poke fun at me than I pay them back in the same way—but more so.	<0.001	<0.001	0.4636	0.35	0.0108	0.0108	0.0001	No
18	If it is for entertaining other people it is justified to make jokes or funny remarks that might be painful or mean about other people.	0.0017	<0.001	<0.001	0.93	0.0016	0.0083	0.0066	No
21	Some people set themselves up for one to make fun at them.	<0.001	<0.001	<0.001	13.39	0.1186	0.1308	0.0122	Yes
24	Since it is only fun, I do not see any problems in compromising others in a funny way.	<0.001	<0.001	<0.001	4.93	0.0178	0.0207	0.0029	No
27	Laughing at others is part of life. People who do not like to be laughed at just should fight back.	0.7573	0.0045	0.0011	0.05	<0.001	0.0017	0.0017	No
30	If I am with a group of people and I am the only one that notices that someone has done something embarrassing or that something embarrassing happened to him/her, than I do not hesitate to tell the others about it.	0.0560	<0.001	<0.001	0.52	<0.001	0.0041	0.0035	No
33	I do not have a bad conscience when I laugh at the misfortunes (e.g., slips of the tongue) of others.	<0.001	<0.001	<0.001	7.95	0.1194	0.1264	0.0070	Yes
36	Nothing is better than stealing a pretenders thunder with a funny remark.	<0.001	<0.001	<0.001	0.91	0.0239	0.0310	0.0071	No
39	It is easier for me to laugh at others than to make fun of myself.	<0.001	<0.001	<0.001	0.45	0.0089	0.0139	0.0050	No
42	In my circle of friends I am known for my ‘‘sharp tongue’’ (e.g., making cynical remarks and jokes about others).	<0.001	<0.001	<0.001	0.54	0.0092	0.0111	0.0019	No
45	I, myself notice that I sometimes cross the line and jokes that others experience as painful started harmless (at least from the viewpoint of demure people).	<0.001	<0.001	<0.001	8.78	0.0320	0.0416	0.0096	Yes

*Note.* χ ^12^, χ ^23^, and χ ^13^ represents the χ ^2^ likelihood ratio statistic between Models 1 and 2, Models 2 and 3, and Models 1 and 3, respectively. *R*_12_, *R*_23_, and *R*_13_ represent McFadden’s pseudo *R*^2^ from a comparison Models 1 and 2, Models 2 and 3, and Models 1 and 3, respectively; Δβ represents the change in β as a percentage

## Data Availability

The data presented in this study are available on request from the corresponding author. The data are not publicly available.

## Appendix A

**Table A1:** Gelotophobia item response theory parameters for the Canadian English version.

Item	F1	S_X2 (df)	a	b_1_	b_2_	b_3_
1. When they laugh in my presence I get suspicious.	0.53	80.77 (73)	1.06	−1.52	0.6	3.1
4. I avoid displaying myself in public because I fear that people could become aware of my insecurity and could make fun of me.	0.68	62.2 (64)	1.58	−1.14	−0.21	1.81
7. When strangers laugh in my presence I often relate it to me personally.	0.54	95.7 (71)	1.10	−1.48	0.46	2.75
10. When others make joking remarks about me I feel being paralyzed.	0.71	70.13 (61)	1.73	−0.82	0.83	2.5
16. I control myself strongly in order not to attract negative attention so I do not make a ridiculous impression.	0.41	101.46 (76)	0.77	−3.86	−1.05	2.18
19. When I have made an embarrassing impression somewhere, I avoid the place thereafter.	0.59	110.88 (67)	1.26	−2.29	−0.43	1.82
22. Some people set themselves up for one to make fun at them.	0.53	85.31 (74)	1.06	−1.23	1.13	3.29
25. It takes me very long to recover from having been laughed at.	0.71	47.68 (62)	1.72	−0.74	0.79	2.22
28. Especially when I feel relatively unconcerned, the risk is high for me to attract negative attention and appear peculiar to others.	0.44	81.09 (74)	0.84	−1.84	1.13	4.10
31. It is difficult for me to hold eye contact because I fear being assessed in a disparaging way.	0.63	65.0 (67)	1.37	−0.89	0.50	1.98
34. Although I frequently feel lonely, I have the tendency not to share social activities in order to protect myself from derision.	0.63	68.71 (66)	1.39	−1.04	0.63	2.32
37. When I have made a fool of myself in front of others I grow completely stiff and lose my ability to behave adequately.	0.67	63.07 (64)	1.55	−0.94	0.74	2.63
40. While dancing I feel uneasy because I am convinced that those watching me assess me as being ridiculous.	0.47	63.45 (77)	0.91	−1.86	−0.25	1.57
43. If I did not fear making a fool of myself I would speak much more in public.	0.52	76.98 (72)	1.02	−2.31	−0.54	1.36

**Table A2:** Gelotophilia item response theory parameters for the Canadian English version.

Item	F1	S_X2 (df)	a	b_1_	b_2_	b_3_
2. When I am with other people, I enjoy making jokes at my own expense to make the others laugh.	0.62	80.39 (69)	1.35	−2.63	−0.97	1.05
5. I do not hesitate telling friends or acquaintances something embarrassing or a misfortune that happened to me, even at the risk of being laughed at.	0.69	65.24 (64)	1.62	−2.40	−1.05	0.62
8. There is no difference for me whether people laugh at me or laugh with me.	0.45	102.66 (78)	0.85	−1.2	1.14	3.45
11. I enjoy it if other people laugh at me.	0.60	89.81 (68)	1.29	−1.0	0.97	2.98
14. I am the joker in my circle of friends, who entertains the others (often with jokes at my own expense).	0.58	60.56 (72)	1.22	−1.92	−1.10	1.97
17. I enjoy it if other people poke fun at me since this might also be a sign of recognition.	0.58	68.86 (71)	1.22	−1.74	−0.01	2.55
20. If someone caught me on a camera while something embarrassing or a misfortune happen to me, I would not mind, if s/he send the tape to a television show that broadcast such videos.	0.50	77.56 (76)	0.99	−0.36	1.04	3.25
23. I have talent for being a comedian, cabaret artist or clown.	0.43	91.03 (80)	0.80	−1.52	0.73	3.34
26. For raising laughs I pleasurably make the most out of embarrassments or misfortunes that happen to me which other people would be ashamed of.	0.74	53.61 (59)	1.87	−1.55	−0.19	1.56
29. I enjoy contributing to the open laughter of others by telling them embarrassing things or misfortunes that happened to me.	0.80	70.03 (52)	2.28	−2.0	−0.82	0.96
32. When I am with other people and something embarrassing happens to me (e.g., a slip of the tongue or a misfortune) I am more pleased than angry and laugh along with it.	0.58	78.63 (70)	1.22	−2.82	−1.0	1.41
35. If I drop a clanger, I enjoy it a little because I can hardly wait to tell my friends about this misfortune.	0.62	72.32 (68)	1.33	−2.22	−0.31	2.0
38. I do not mind telling something embarrassing in a group that happened to me if I know that the others will find it funny.	0.76	72.86 (55)	1.96	−2.60	−1.30	0.57
41. Nothing much could happen to me that I would be so ashamed that I would not tell it others.	0.48	85.74 (78)	0.94	−1.7	0.36	2.84
44. My friends know me for not being ashamed of telling them of embarrassing situations that happened to me.	0.69	96.57 (62)	1.64	−1.96	−0.49	1.24

**Table A3:** Katagelasticism item response theory parameters for the Canadian English version.

Item	F1	S_X2 (df)	a	b_1_	b_2_	b_3_
3. I enjoy exposing others and I am happy when they get laughed at.	0.70	76.12 (65)	1.68	−0.42	0.89	2.39
6. Often, disputes emerged because of funny remarks or jokes that I make about other people.	0.69	68.69 (61)	1.61	−0.17	1.29	2.73
9. When related to making jokes or funny remarks about other people I rather follow the motto ‘‘An eye for an eye, a tooth for a tooth’’ than ‘‘If someone strikes you on the left cheek, offer him the other also.’’	0.43	110.14 (78)	0.81	−2.35	0.14	3.10
12. It has happened that humorless persons have broken o¤ their friendship with me or at least threatened me to do so, because I overdid ridiculing them over of something embarrassing or a misfortune that happened to them.	0.61	85.38 (65)	1.32	0.46	1.76	3.22
15. If other people poke fun at me than I pay them back in the same way—but more so.	0.60	79.07 (69)	1.29	−1.53	0.35	2.55
18. If it is for entertaining other people it is justified to make jokes or funny remarks that might be painful or mean about other people.	0.78	75.68 (55)	2.14	−0.12	0.96	2.35
21. Some people set themselves up for one to make fun at them.	0.48	100.0 (76)	0.92	−2.70	−0.86	2.20
24. Since it is only fun, I do not see any problems in compromising others in a funny way.	0.76	74.52 (59)	2.02	−0.79	0.64	2.33
27. Laughing at others is part of life. People who do not like to be laughed at just should fight back.	0.65	80.93 (68)	1.45	−1.33	0.50	2.34
30. If I am with a group of people and I am the only one that notices that someone has done something embarrassing or that something embarrassing happened to him/her, than I do not hesitate to tell the others about it.	0.63	74.32 (69)	1.37	−0.98	0.76	2.74
33. I do not have a bad conscience when I laugh at the misfortunes (e.g., slips of the tongue) of others.	0.35	109.39 (80)	0.63	−3.76	−0.65	2.86
36. Nothing is better than stealing a pretenders thunder with a funny remark.	0.47	102.34 (74)	0.92	−2.15	0.48	2.99
39. It is easier for me to laugh at others than to make fun of myself.	0.35	117.49 (82)	0.64	−3.24	0.22	3.53
42. In my circle of friends I am known for my ‘‘sharp tongue’’ (e.g., making cynical remarks and jokes about others).	0.56	72.01 (75)	1.15	−1.17	0.66	2.34
45. I, myself notice that I sometimes cross the line and jokes that others experience as painful started harmless (at least from the viewpoint of demure people).	0.59	65.64 (72)	1.25	−1.09	0.32	2.26

**Table A4:** Gelotophobia item response theory parameters for the Taiwanese Chinese version.

Item	F1	S_X2 (df)	a	b1	b2	b3
1. When they laugh in my presence I get suspicious.	0.52	97.67	1.05	−2.88	0.16	3.58
4. I avoid displaying myself in public because I fear that people could become aware of my insecurity and could make fun of me.	0.69	49.68	1.63	−2.178	−0.07	1.99
7. When strangers laugh in my presence I often relate it to me personally.	0.56	59.51	1.15	−1.62	1.00	3.83
10. When others make joking remarks about me I feel being paralyzed.	0.61	70.94	1.3	−2.07	0.87	3.27
16. I control myself strongly in order not to attract negative attention so I do not make a ridiculous impression.	0.62	66.54	1.34	−2.3	−0.02	2.46
19. When I have made an embarrassing impression somewhere, I avoid the place thereafter.	0.63	59.20	1.05	−2.88	0.01	2.84
22. Some people set themselves up for one to make fun at them.	0.53	46.91	1.08	−1.57	2.25	5.34
25. It takes me very long to recover from having been laughed at.	0.54	58.27	1.35	−1.25	1.38	3.33
28. Especially when I feel relatively unconcerned, the risk is high for me to attract negative attention and appear peculiar to others.	0.62	87.02	0.81	−2.28	1.67	5.25
31. It is difficult for me to hold eye contact because I fear being assessed in a disparaging way.	0.43	58.35	1.61	−0.94	1.22	2.82
34. Although I frequently feel lonely, I have the tendency not to share social activities in order to protect myself from derision.	0.69	49.49	1.82	−0.78	1.28	2.94
37. When I have made a fool of myself in front of others I grow completely stiff and lose my ability to behave adequately.	0.73	65.97	1.74	−1.99	0.19	2.48
40. While dancing I feel uneasy because I am convinced that those watching me assess me as being ridiculous.	0.72	70.82	1.05	−2.3	−0.08	2.43
43. If I did not fear making a fool of myself I would speak much more in public.	0.52	61.57	1.91	−1.41	0.54	2.36

**Table A5:** Gelotophilia item response theory parameters for the Taiwanese Chinese version.

Item	F1	S_X2 (df)	a	b1	b2	b3
2. When I am with other people, I enjoy making jokes at my own expense to make the others laugh.	0.66	50.78	1.52	−2.93	−0.37	2.27
5. I do not hesitate telling friends or acquaintances something embarrassing or a misfortune that happened to me, even at the risk of being laughed at.	0.66	44.63	1.49	−3.14	−1.37	1.08
8. There is no difference for me whether people laugh at me or laugh with me.	0.34	79.93	0.62	−3.41	0.86	6.00
11. I enjoy it if other people laugh at me.	0.53	62.90	1.05	−2.61	0.57	4.14
14. I am the joker in my circle of friends, who entertains the others (often with jokes at my own expense).	0.64	54.02	1.41	−2.86	−3.34	2.06
17. I enjoy it if other people poke fun at me since this might also be a sign of recognition.	0.56	62.19	1.16	−2.86	−0.07	3.54
20. If someone caught me on a camera while something embarrassing or a misfortune happen to me, I would not mind, if s/he send the tape to a television show that broadcast such videos.	0.35	64.31	0.64	−1.42	2.41	6.91
23. I have talent for being a comedian, cabaret artist or clown.	0.53	61.89	1.07	−2.35	0.28	3.03
26. For raising laughs I pleasurably make the most out of embarrassments or misfortunes that happen to me which other people would be ashamed of.	0.65	47.46	1.45	−2.74	−0.27	2.45
29. I enjoy contributing to the open laughter of others by telling them embarrassing things or misfortunes that happened to me.	0.83	51.98	−2.55	2.08	−0.51	1.72
32. When I am with other people and something embarrassing happens to me (e.g., a slip of the tongue or a misfortune) I am more pleased than angry and laugh along with it.	0.61	44.50	1.30	−3.87	−1.65	1.47
35. If I drop a clanger, I enjoy it a little because I can hardly wait to tell my friends about this misfortune.	0.77	45.79	2.08	−2.10	−0.40	1.53
38. I do not mind telling something embarrassing in a group that happened to me if I know that the others will find it funny.	0.80	58.36	2.24	−2.48	−1.04	1.37
41. Nothing much could happen to me that I would be so ashamed that I would not tell it others.	0.68	49.80	1.57	−2.64	−0.06	2.05
44. My friends know me for not being ashamed of telling them of embarrassing situations that happened to me.	0.43	153.24	0.82	−3.53	−0.33	3.24

**Table A6:** Katagelasticism item response theory parameters for the Taiwanese Chinese version.

Item	F1	S_X2 (df)	a	b_1_	b_2_	b_3_
3. I enjoy exposing others and I am happy when they get laughed at.	0.76	39.52	2.0	−0.58	1.24	2.96
6. Often, disputes emerged because of funny remarks or jokes that I make about other people.	0.70	45.05	1.69	−0.71	1.51	3.43
9. When related to making jokes or funny remarks about other people I rather follow the motto ‘‘An eye for an eye, a tooth for a tooth’’ than ‘‘If someone strikes you on the right cheek, offer him the other also.’’	0.51	102.70	1.01	−2.01	0.38	2.93
12. It has happened that humorless persons have broken o¤ their friendship with me or at least threatened me to do so, because I overdid ridiculing them over of something embarrassing or a misfortune that happened to them.	0.44	54.69	0.83	0.20	3.20	6.19
15. If other people poke fun at me than I pay them back in the same way—but more so.	0.55	69.24	1.12	−1.31	1.33	3.49
18. If it is for entertaining other people it is justified to make jokes or funny remarks that might be painful or mean about other people.	0.63	62.77	1.38	−0.45	1.77	3.94
21. Some people set themselves up for one to make fun at them.	0.78	56.53	2.12	−0.79	1.26	2.84
24. Since it is only fun, I do not see any problems in compromising others in a funny way.	0.62	92.68	1.35	−1.67	0.54	3.25
27. Laughing at others is part of life. People who do not like to be laughed at just should fight back.	0.55	60.81	1.13	−1.78	0.93	3.85
30. If I am with a group of people and I am the only one that notices that someone has done something embarrassing or that something embarrassing happened to him/her, than I do not hesitate to tell the others about it.	0.53	58.26	1.06	−1.69	1.36	4.05
33. I do not have a bad conscience when I laugh at the misfortunes (e.g., slips of the tongue) of others.	0.58	47.34	1.23	−0.57	2.16	3.88
36. Nothing is better than stealing a pretenders thunder with a funny remark.	0.75	70.88	1.92	−0.90	1.20	2.80
39. It is easier for me to laugh at others than to make fun of myself.	0.61	60.04	1.31	−1.59	0.78	3.19
42. In my circle of friends I am known for my ‘‘sharp tongue’’ (e.g., making cynical remarks and jokes about others).	0.64	84.17	1.41	−0.52	1.21	1.98
45. I, myself notice that I sometimes cross the line and jokes that others experience as painful started harmless (at least from the viewpoint of demure people).	0.32	115.56	0.58	−4.34	−0.98	3.68
